# Reconstruction method to combine high temporal resolution with appropriate image quality in dynamic PET angiography

**DOI:** 10.1007/s00259-020-04962-0

**Published:** 2020-07-23

**Authors:** Christian Kühnel, Philipp Seifert, Robert Drescher, Martin Freesmeyer

**Affiliations:** Clinic of Nuclear Medicine, Jena University Hospital, Am Klinikum 1, 07747 Jena, Germany

Dear Sir,

With great interest, we read the Image of the Month article by Norikane et al., “One-stop shopping 18F-FDG PET/CT in a patient with vascular type Behcet’s disease” [1]. They describe the application of PET angiography to depict venous vascular malformations. The images shown are of outstanding quality, clearly depicting the vascular anatomy and hemodynamics in the first 21 s after tracer injection.

PET angiography is based on a continuous, dynamic PET acquisition starting simultaneously with tracer injection [2]. Images are reconstructed retrospectively from the list-mode raw data in user-selected time intervals (frames), which may range from few seconds to several minutes [3].

When considering the length of frames which should be reconstructed, a tradeoff has to be made between temporal resolution and signal-to-noise ratio of the resulting image sequence. A shorter frame represents a shorter snapshot of the hemodynamic evolvement of the tracer bolus in the vasculature, but image quality may be limited due to poor count statistics. A longer frame contains more PET data for reconstruction, leading to higher image quality for visualization of detail. However, the dynamic information is averaged over the length of the frame.

Norikane et al. were able to achieve high image quality in consecutive 3-s frames because a venous circulation was depicted after intravenous injection of the radiotracer. For PET angiography of arteries, a relevant dilution of the tracer bolus occurs in the cardiopulmonary circulation until it reaches the target vessels [4]. Frame lengths of at least 7–10 s are necessary.

An option to combine high temporal resolution with appropriate image quality in dynamic PET angiography, particularly for an animated display of the findings, is to reconstruct interleaved frames from the PET data, while preserving frame duration. In the presented case, 7-s frames were reconstructed with a 1 s time offset, e.g., 0–7 s, 1–8 s, 2–9 s, and so on (matrix 400, iterations 4, subsets 12 and Gaussian filter FWHM 5; performed with HD TrueX software, Siemens Healthineers, Erlangen, Germany). Each resulting image contains the averaged temporal information of 7 s of blood flow. The images are merged into a new sequence. A frame length of 7 s ensures sufficient image quality, and the short intervals of 1 s avoid loss of temporal information which would happen with the standard reconstruction method (Fig. 1). Visual smoothness of blood flow depiction is improved (Suppl. 1).

**Fig. 1:**
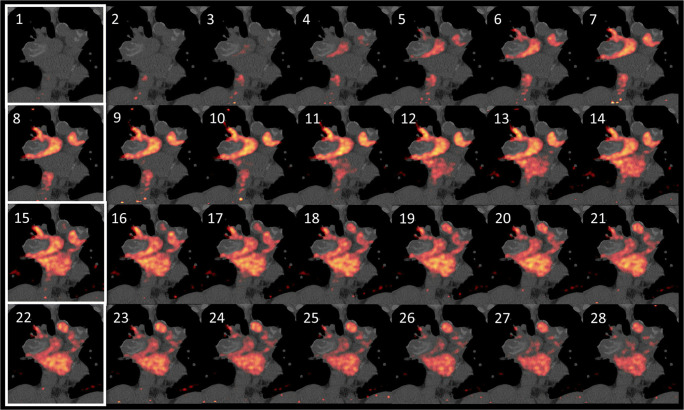
Image sequence of interleaved reconstruction. Interval between images is 1 s. A conventional reconstruction of the same data would only consist of images 1, 8, 15, and 22 (white margins). Wall-adherent, calcified thrombi are seen in the pulmonary arteries

The described “reframing” method requires a significant technical effort, but may be helpful for specific cases with complicated vascular malformations, for demonstration purposes, and in patients with contraindications to other imaging methods.

## Electronic supplementary material

ESM 1 Video sequences of conventional and interleaved image reconstructions. The temporal resolution of the interleaved sequence is higher, without losing image quality. (WMV 1559 kb)
